# Einflussfaktoren beim Aufbau von Präventionsketten in Neubaugebieten am Beispiel des Münchner Stadtteils Freiham – eine qualitative Studie

**DOI:** 10.1007/s11553-022-01001-8

**Published:** 2022-12-12

**Authors:** Stephan Voss, Michaela Coenen, Julia Hummel, Caroline Jung-Sievers, Valerie Zu Rhein, Eva Rehfuess

**Affiliations:** 1 Institut für Medizinische Informationsverarbeitung, Biometrie und Epidemiologie – IBE, Lehrstuhl für Public Health und Versorgungsforschung, Ludwig-Maximilians-Universität München, Elisabeth-Winterhalter-Weg 6, 81377 München, Deutschland; 2 Pettenkofer School of Public Health, München, Deutschland

**Keywords:** Kommunale Gesundheitsförderung, Präventionskette, Intersektorales Netzwerk, Kinder- und Jugendgesundheit, Gesundheitliche Ungleichheit, Interviewstudie, Prozessevaluation, Community health promotion, “Präventionsketten”, Intersectoral network, Child and adolescent health, Health inequality, Interview study, Process evaluation

## Abstract

**Einleitung:**

Präventionsketten sind integrierte kommunale Strategien zur Förderung von Gesundheit und sozialer Teilhabe von Kindern, Jugendlichen und Familien sowie zur Vermeidung der Folgen von Kinderarmut. Sie sind als intersektorale Netzwerke in mehreren Kommunen in Deutschland etabliert. Der Aufbau von Präventionsketten in Neubaugebieten wurde bislang noch nicht systematisch erforscht. Im Rahmen der Prozessevaluation zur Präventionskette Freiham, die in dem sich derzeit im Bau befindlichen gleichnamigen Münchner Stadtteil implementiert wird, wurde eine qualitative Interview- und Fokusgruppenstudie durchgeführt. Das Ziel war, relevante Einflussfaktoren für den Aufbau von Präventionsketten in einem neu entstehenden Stadtteil zu identifizieren.

**Methoden:**

Für die Studie wurden wiederholt Interviews mit dem Netzwerkwerkmanagement der Präventionskette Freiham durchgeführt sowie eine Fokusgruppe mit Vertreter*innen der beteiligten städtischen Referate. Zudem fanden Interviews mit 12 lokalen Fachkräften aus den Sektoren Bildung, Soziales und Gesundheit statt. Die Auswertung erfolgte mit der Methode der qualitativen Inhaltsanalyse in Anlehnung an Mayring.

**Ergebnisse:**

Wichtige Einflussfaktoren für eine gelingende Implementierung stellten aus Sicht der Teilnehmer*innen die Ausstattung mit finanziellen und personellen Ressourcen sowie die Unterstützung durch die kommunale Politik und Verwaltungsspitzen dar. Für eine erfolgreiche Arbeit im Netzwerk waren die zentrale Netzwerkkoordination, eine transparente Kommunikation, die Integration der unterschiedlichen Interessen der Akteur*innen und die Vermittlung eines Mehrwerts des Engagements entscheidend. Eine spezifische Herausforderung für das Setting eines Neubaugebiets war der Aufbau von bedarfs- und zielgruppenorientierten Netzwerkstrukturen angesichts einer zunächst geringen Anzahl an Bewohner*innen und noch nicht etablierter Unterstützungsstrukturen.

**Schlussfolgerung:**

Ausreichende Finanzierung und Rückhalt in Politik und Verwaltung sind zentrale Einflussfaktoren für die Implementierung einer Präventionskette in Kommunen. Insbesondere in Neubaugebieten ohne gewachsene Infrastruktur erfordert der Aufbauprozess eine langfristig angelegte Unterstützung.

## Abstract (en)

**Introduction:**

“Präventionsketten” (prevention chains) are integrated municipal strategies to promote health and social participation for children, adolescents, and families and to prevent the negative consequences of child poverty. They exist as intersectoral networks in multiple communities in Germany. However, the implementation of such intersectoral networks in new residential development areas has not yet been systematically examined. As part of the process evaluation of the intervention “Präventionskette Freiham”, which is being implemented in the Munich district of Freiham, a qualitative interview and focus group study was conducted. The aim was to identify relevant influencing factors for the implementation of this integrated municipal strategy.

**Methods:**

For this study, repeated interviews were conducted with the network management of the “Präventionskette”, and a focus group was held with representatives of the involved municipal departments. Furthermore, interviews were undertaken with 12 professionals working locally in the education, social and health sectors. The data were analyzed using qualitative content analysis following Mayring.

**Results:**

According to participants, general key influencing factors for a successful implementation were the provision of financial and human resources as well as the support from local policy makers and high-level administrators. The network coordination, transparent communication, the integration of the interests of different network members, and a clear communication of the added value of engaging with the network were central factors for successful operations. Regarding the specific setting of a new residential development area, implementing demand-oriented network structures with only a small number of initial residents and a still-to-be-established supporting infrastructure presented challenges.

**Conclusion:**

Sufficient funding and commitment by policy makers and administrators are central influencing factors for the implementation of “Präventionsketten” in municipalities. Especially in a new residential development area without an established infrastructure, the development process requires a long-term commitment.

## Einleitung

Gesundheitliche Nachteile für Kinder mit einem niedrigen sozioökonomischen Hintergrund sind für Deutschland gut belegt [19, 35]. In der KiGGS-Studie empfanden Kinder aus Familien mit einem niedrigen sozioökonomischen Status ihren Gesundheitszustand im Durchschnitt als schlechter wie ihre Altersgenoss*innen und litten eher unter chronischen Erkrankungen wie Adipositas sowie unter eingeschränkter mentaler Gesundheit [18]. Außerdem zeigten sie häufiger gesundheitsschädliche Verhaltensweisen wie eine geringe körperliche Aktivität, eine ungesunde Ernährung und Tabakkonsum [18]. Sozial benachteiligte Familien nehmen zudem seltener Angebote zur Gesundheitsförderung in Anspruch [8]. Gesundheitliche Ungleichheiten bei Kindern wirken sich bis ins Erwachsenenalter aus [23]. Ansätze zur Prävention der gesundheitlichen Folgen von Kinderarmut sind deswegen aus Public-Health-Sicht als besonders vielversprechend anzusehen, da sie das Potenzial haben, jahrzehntelange Krankheitslast vorzubeugen [29].

Sektorenübergreifende Strategien zur Gesundheitsförderung von Kindern und Jugendlichen im kommunalen Setting sowie zur Bekämpfung der Folgen von Kinderarmut sind in Deutschland als Präventionsketten bekannt [27, 28]. In Präventionsketten stehen Kinder und ihre Familien sowie die Bedürfnisse dieser Zielgruppen im Fokus [27]. Dabei verfolgen sie einen ganzheitlichen, sektorenübergreifenden Ansatz. Präventionsketten setzen auf eine engere Vernetzung von Akteur*innen, die für das Kindeswohl in der Kommune zuständig sind, um eine bessere Abstimmung der unterschiedlichen Angebote für Kinder und Familien zu erreichen. Diese Vernetzung erfolgt einerseits horizontal, zwischen den verschiedenen Sektoren wie Bildung und Gesundheit. Gleichzeitig erfolgt sie vertikal, also über mehrere Hierarchieebenen hinweg und zwischen den direkt mit den Zielgruppen befassten Fachkräften auf der einen Seite und der kommunalen Verwaltung auf der anderen Seite. Letzterer kommt bei der Planung und Durchführung von Präventionsketten eine Schlüsselrolle hinsichtlich der Steuerung der Vernetzungs- und Abstimmungsprozesse zu [27]. Zudem ist die Arbeit von Präventionsketten in der Regel lebensphasen- und lebenslagenübergreifend ausgerichtet. Gerade die Übergänge zwischen den einzelnen Abschnitten im Leben eines Kindes, etwa vom Kindergarten in die Grundschule, gelten als eine besondere Herausforderung. Werden sie erfolgreich gemeistert, können sie ressourcenstärkend wirken. Im ungünstigen Fall können sie dagegen Risikofaktoren für die Entwicklung darstellen [28].

Erfahrungen mit dem Aufbau und der Umsetzung von Präventionsketten haben bereits mehrere Kommunen in Deutschland gesammelt – etwa beim Projekt „Präventionsketten Niedersachsen: Gesund aufwachsen für alle Kinder“ [22] oder im Modellprojekt „Kommunale Präventionsketten“ (ehemals „Kein Kind zurücklassen – KeKiz“) in Nordrhein-Westfalen (NRW) [3]. In der bayerischen Landeshauptstadt München wird mit der Präventionskette Freiham „Gut und gesund aufwachsen“, die im Stadtteil Freiham parallel zum Aufbau des Stadtteils implementiert wird, zum ersten Mal eine solche kommunale Gesamtstrategie zur Gesundheitsförderung von Kindern und Jugendlichen verfolgt. Der Stadtteil Freiham entsteht derzeit im Westen der Stadt München in zwei Realisierungsabschnitten. Ende 2019 zogen die ersten Bewohner*innen ein. Nach Fertigstellung beider Abschnitte, etwa um das Jahr 2040, sollen rund 25.000 Menschen in dem Gebiet wohnen [20]. Bereits im Jahr 2015 initiierte die Stadt München den Aufbau einer Präventionskette für den neuen Stadtteil. Das Ziel ist eine engere Kooperation der drei beteiligten kommunalen Referate – dem Referat für Bildung und Sport, dem Gesundheitsreferat und dem Sozialreferat – sowie eine bessere Vernetzung der lokalen Akteur*innen, um in Freiham von Beginn an gesundheitliche Chancengleichheit und soziale Teilhabe für benachteiligte Kinder und Familien zu fördern. Der Aufbau eines lokalen Netzwerks im Quartier wurde Anfang 2020 intensiviert, kurz nach Einzug der ersten Bewohner*innen. Eine Besonderheit der Präventionskette Freiham ist, dass in diesem Fall das Netzwerk nicht in einem Bestandsgebiet mit gewachsenen Strukturen implementiert wird, sondern die fachgebietsübergreifende Vernetzung von Beginn an bei der Entstehung der Infrastruktur berücksichtigt werden soll [21]. Der Aufbau des Netzwerks im Stadtteil wird von einem Team am Lehrstuhl für Public Health und Versorgungsforschung der Ludwig-Maximilians-Universität München wissenschaftlich begleitet und evaluiert.

Präventionsketten wurden deutschlandweit bereits in mehreren Kommunen implementiert. Ihre wissenschaftliche Erforschung ist aber nach wie vor selten [15]. Zu den Ausnahmen zählen hier die Evaluation des Projekts „Kommunale Präventionsketten“ [3] und die Evaluation zum Präventionsnetzwerk Ortenaukreis [14]. Ein wichtiger Grund für den Mangel an Forschung ist dabei, dass es sich bei Präventionsketten als Maßnahmen zur Gesundheitsförderung im Setting der Kommune um komplexe Interventionen handelt, deren Evaluation methodisch anspruchsvoll und entsprechend aufwändig ist [6, 15]. Eine Herausforderung bei der Erforschung der Wirksamkeit von Präventionsketten und vergleichbaren Maßnahmen ist, dass die Outcomes oft schwer zu operationalisieren sind, Effekte erst nach längerer Zeit zu erwarten sind und eine Attribution dieser Effekte direkt auf die Intervention aufgrund der Komplexität des Settings Kommune kaum möglich ist [15]. Als Folge gibt es national wie international wenig Evidenz zur Wirkung von kommunalen Maßnahmen zur Verbesserung der Gesundheit und zur Verringerung von gesundheitlichen Ungleichheiten [26]. Umso mehr gewinnt bei der Erforschung entsprechender Maßnahmen die Untersuchung der Implementierung an Bedeutung – sog. Prozessevaluationen [25]. Prozessevaluationen ermöglichen ein besseres Verständnis davon, wie eine Intervention mit ihrem Kontext interagiert und welche Faktoren zu Erfolg oder Misserfolg beitragen. Das erlaubt einerseits eine bestehende Maßnahme oder ihre Implementierung zu modifizieren und andererseits die Übertragung einer Maßnahme und ihrer Umsetzung in einen neuen Kontext zu verbessern [11].

Unseres Wissens nach liegt noch keine Evaluation der Implementierung einer Präventionskette in einem Neubaugebiet vor. Das Ziel der vorliegenden Arbeit ist es, anhand der Präventionskette Freiham relevante Einflussfaktoren beim Aufbau einer Präventionskette zu erforschen, mit besonderem Bezug auf das spezifische Setting eines im Bau befindlichen Stadtteils. Dazu werden Daten von qualitativen Interviews und einer Fokusgruppe ausgewertet, die im Rahmen einer umfassenden Prozessevaluation des Projekts mit Stakeholdern der Präventionskette Freiham geführt wurden.

## Methodik

### Die Präventionskette Freiham

**Abb. 1:**
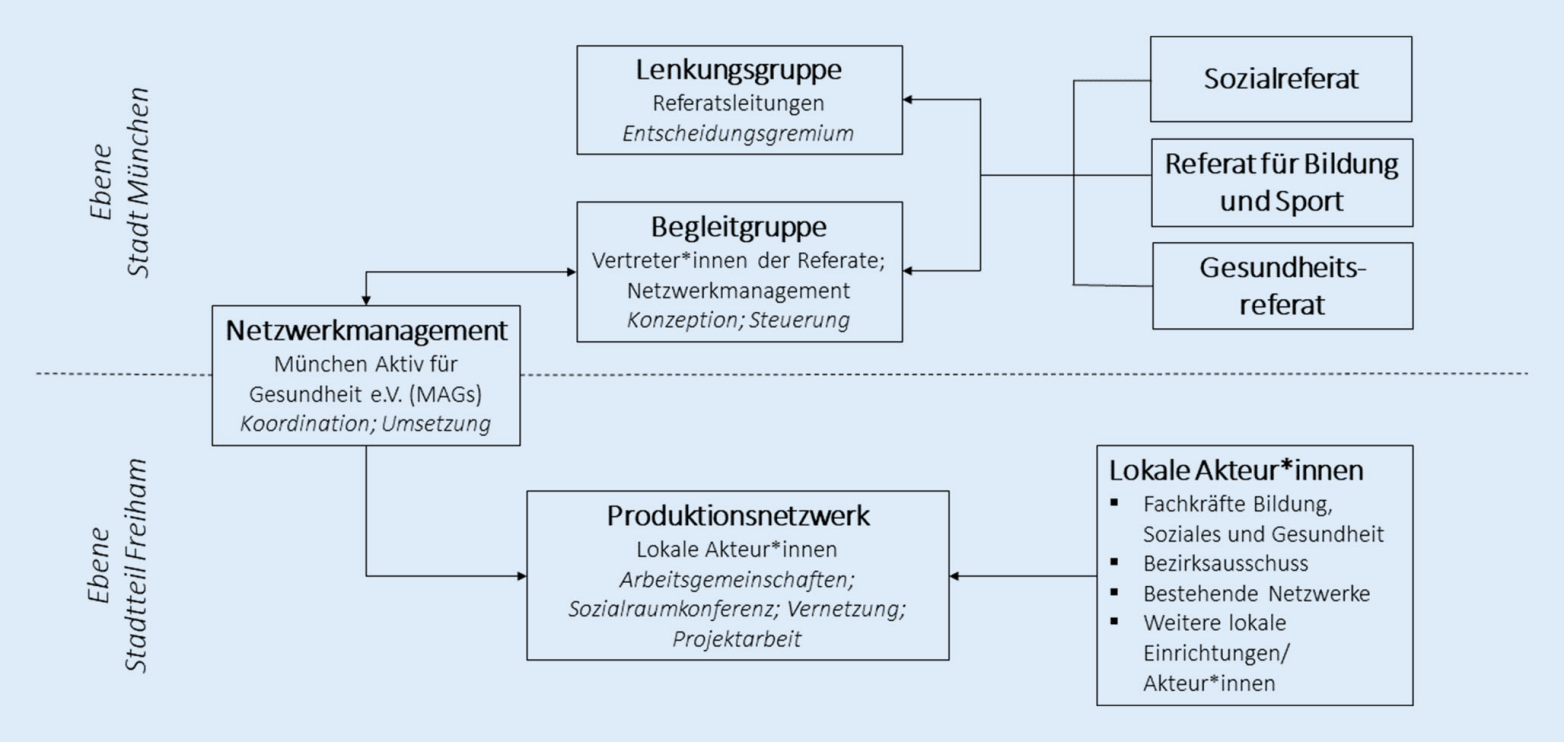
Struktur der Präventionskette Freiham

Das Ziel der Präventionskette Freiham ist eine Vernetzung der Angebotsinfrastruktur für Kinder, Jugendliche und Familien im neuen Münchner Stadtteil Freiham, um die gesundheitliche Chancengleichheit und soziale Teilhabe für alle Heranwachsenden im Stadtteil zu fördern und den Folgen von Kinderarmut vorzubeugen. Die Basis bildet die durch einen Kooperationsvertrag verbindlich geregelte Zusammenarbeit von drei kommunalen Referaten der Stadt München: dem Referat für Bildung und Sport, dem Gesundheitsreferat (bis 2020 Referat für Gesundheit und Umwelt) sowie dem Sozialreferat. Die Präventionskette setzt sich aus mehreren Kernelementen zusammen (Abb. 1): (a) Die *Lenkungsgruppe* bildet das oberste Entscheidungsgremium, das aus den Leitungen bzw. Stellvertretungen der drei beteiligten Referate besteht. (b) Die regelmäßig tagende *Begleitgruppe* fungiert als Konzeptions- und Steuerungsgremium, in dem Vertreter*innen der drei Referate sowie das Netzwerkmanagement sitzen. (c) Das *Netzwerkmanagement* der Präventionskette, das für den Aufbau und die Organisation des Netzwerks im Stadtteil zuständig ist und als Transmissionsriemen zwischen Stadtteil und kommunaler Verwaltung sowie der Begleitgruppe dient. Das Netzwerkmanagement der Präventionskette Freiham besteht seit 2016 und wird seitdem vom freien Träger München Aktiv für Gesundheit e. V. (MAGs) übernommen. (d) Das lokale *Produktionsnetzwerk*, das v. a. aus Fachkräften aus den Bereichen Bildung, Soziales und Gesundheit besteht, hat seinen Arbeitsbereich vor Ort im Stadtteil Freiham. Kern des Produktionsnetzwerks bilden die nach Alter der Zielgruppen gestaffelten Arbeitsgemeinschaften, in denen die Akteur*innen sich regelmäßig treffen und austauschen.

### Studiendesign

Die Evaluation der Präventionskette Freiham erfolgt durch den Lehrstuhl für Public Health und Versorgungsforschung an der Ludwig-Maximilians-Universität München. Im Rahmen der Projektphase der Prozessevaluation (Laufzeit: November 2019–Dezember 2021, Förderung durch die Bundeszentrale für gesundheitlich Aufklärung, BZgA) wurden – neben weiteren Untersuchungen – in 3 Teilstudien qualitative Datenerhebungen mit Personengruppen durchgeführt, die für die Implementierung des Projekts relevant sind:

- Wiederholte Einzelinterviews mit dem Netzwerkmanagement der Präventionskette Freiham im Abstand von jeweils 3 bis 4 Monaten (April 2020–Oktober 2021). Das Ziel der wiederholten Befragungen war, einen detaillierten Einblick in die Ansichten des Netzwerkmanagements zur Entwicklung des Netzwerkaufbaus über den Zeitraum der Evaluation zu erhalten.
- Eine Fokusgruppe mit Vertreter*innen der drei städtischen Referate aus der Begleitgruppe der Präventionskette (Februar 2021).
- Einzelinterviews mit lokalen Fachkräften, in deren Zuständigkeitsbereich Freiham zum Zeitpunkt der Datenerhebung lag. Teilnehmer*innen wurden dabei nach Möglichkeit jeweils 2‑mal in zwei Erhebungswellen (Oktober–Dezember 2020; Juni–Juli 2021) interviewt. Hier war das Ziel der wiederholten Befragungen, die Perspektive der Fachkräfte auf den Vernetzungsprozess zu unterschiedlichen Zeitpunkten zu erfassen und den Teilnehmer*innen die Gelegenheit zu geben, im zweiten Interview über ihre Ansichten aus der ersten Erhebung zu reflektieren.

Der Schwerpunkt der Interviews und der Fokusgruppe lag darauf, die jeweilige Sicht der Beteiligten auf den laufenden Implementierungsprozess des Netzwerks in Freiham sowie auf dabei relevante Einflussfaktoren zu erforschen. Durch die Befragung der drei unterschiedlichen Personengruppen – Netzwerkmanagement, Begleitgruppe und lokale Fachkräfte – sollte ein möglichst umfassendes Bild auf den Netzwerkaufbau gewonnen werden.

Alle 3 Teilstudien wurden unter Berücksichtigung der ethischen Standards der Deklaration von Helsinki durchgeführt [37]. Allen Teilstudien wurde durch die Ethikkommission der Medizinischen Fakultät an der Ludwig-Maximilians-Universität München die ethisch-rechtliche Unbedenklichkeit erteilt.

### Datenerhebung

Potenzielle Teilnehmer*innen wurden im Vorfeld per E‑Mail vom Interviewer (SV) kontaktiert. Die E‑Mail-Adressen des Netzwerkmanagements sowie der Vertreter*innen der städtischen Referate waren dem Studienteam bekannt. Die Kontakte der lokalen Fachkräfte wurden z. T. über das Netzwerkmanagement als Multiplikator weitergegeben – das Netzwerkmanagement fragte dabei vorab die prinzipielle Teilnahmebereitschaft ab – oder vom Studienteam aus öffentlich zugänglichen Internetauftritten recherchiert. Die Rekrutierung der Fachkräfte erfolgte nach dem Prinzip des *Purposeful Samplings*. Die Teilnehmer*innen sollten nach Möglichkeit bereits an Aktivitäten des Netzwerks teilgenommen haben. Zudem war ein Ziel der Rekrutierungsstrategie, die unterschiedlichen Bereiche der Präventionskette (Bildung, Soziales und Gesundheit) sowie die unterschiedlichen Altersbereiche (0–6 Jahre; 6–17 Jahre) nach Möglichkeit jeweils angemessen abzudecken. Es sollten so viele Interviews durchgeführt werden, bis eine Datensättigung erreicht war und keine wesentlichen weiteren Erkenntnisse mehr zu erwarten waren.

In einer ersten E‑Mail wurden mögliche Teilnehmer*innen jeweils über Inhalte und Ziele der Studie sowie über die Bedingungen zum Datenschutz informiert und das Interesse zur Teilnahme erfragt. Im Falle einer Zusage erhielten sie jeweils die ausführlichen Teilnahmeinformationen zusammen mit den Einwilligungserklärungen per E‑Mail zugesandt. Unmittelbar vor den Interviews und der Fokusgruppe wurden die Teilnehmer*innen erneut ausführlich über die Studienbedingungen und den Datenschutz aufgeklärt. Auf dieser Basis konnten sie final über ihre Teilnahme entscheiden und die Einwilligungserklärungen unterschreiben.

Aufgrund der COVID-19-Pandemie („coronavirus disease 2019“) und den damit einhergehenden Kontaktbeschränkungen fand der Großteil der Datenerhebungen auf digitalen Konferenzplattformen statt – die ersten beiden Interviews mit dem Netzwerkmanagement auf der Open-source-Software Jitsi Meet, ansonsten auf Webex (Cisco Systems, San José, CA, USA). Drei Interviews fanden in Präsenz statt.

Bei den Interviews und der Fokusgruppe kam jeweils ein eigens erstellter semistrukturierter Interviewleitfaden zum Einsatz. Die Leitfäden wurden gemäß der Methode von Helfferich [16] konzipiert anhand der Forschungsfragen, aus bisher veröffentlichter Literatur zu Präventionsketten sowie anhand eines a priori entwickelten logischen Modells zur Präventionskette Freiham. Die Fragen waren offen angelegt, um bei Teilnehmer*innen einen Erzählanreiz zu setzen, bei dem der Interviewer nur zu bestimmten Punkten vertiefende Nachfragen stellte.

Die Gespräche wurden mit der Open-source-Software Audacity digital aufgezeichnet. Anschließend wurden sie mit der Software f4transkript (dr. dresing & pehl GmbH, Marburg, Deutschland) nach den Regeln der einfachen Transkription [7] verschriftlicht. Die Teilnehmer*innen erhielten das fertige Transkript zur Freigabe zugesandt. Namen von Institutionen und Einzelpersonen wurden in den Transkripten pseudonymisiert, indem sie durch Zahlencodes ersetzt wurden.

### Datenauswertung

Die Transkripte wurden nach der Methode der qualitativen Inhaltsanalyse in Anlehnung an Mayring [24] mit der Software MAXQDA (VERBI Software, Berlin, Deutschland) ausgewertet. 3 Autor*innen (JH, SV, VZR) sichteten das vollständige Material. Danach entwickelten sie deduktiv-induktiv ein Hauptkategoriensystem aus den Inhalten der Transkripte und vorherigen theoretischen Überlegungen basierend auf dem logischen Modell. Die 3 Autor*innen wandten das Hauptkategoriensystem jeweils getrennt auf ein Transkript an und verglichen die Ergebnisse, bis das Kategoriensystem nach mehreren Interviews finalisiert wurde und es eine ausreichende Übereinstimmung zwischen den Kodierer*innen gab. Jedes Transkript wurde anschließend von jeweils einer/einem der 3 Autor*innen kodiert. Unstimmigkeiten wurden im Team diskutiert und gemeinsam gelöst. Anschließend entwickelte eine Person für jede Hauptkategorie das Unterkategoriensystem und kodierte alle entsprechenden Textstellen. Die Unterkategorien wurden wieder von einem zweiten Teammitglied gesichtet und Unstimmigkeiten in der Gruppe geklärt. Für die Kodierung kam ein Kodierleitfaden zum Einsatz, in dem für alle Kategorien Hinweise zum Kodieren sowie mindestens ein Ankerbeispiel beschrieben wurden. Ein Auszug ist in Tab. 1 zu finden.

**Tab. 1:** Auszug aus dem Kodierleitfaden

Bezeichnung Hauptkategorie	Bezeichnung Unterkategorie	Kodierhinweis	Ankerbeispiel(e)
Netzwerkmitglieder	–	Kodiere alle Stellen, in denen die Interviewten darüber sprechen, wie die Mitglieder des Netzwerks Einfluss auf Verlauf und Effektivität des Projekts nehmen bzw. welche Faktoren Einfluss auf Mitarbeit der Netzwerkmitglieder nehmen	*„Es gibt eine Hemmung von der Bearbeitung der gemeinsamen Fälle. Da konnte man natürlich, das müsste man herausstellen, welchen Nutzen, also, wir müssten mal einen gemeinsamen Fall bearbeiten und welchen Nutzen hätte es bringen können?“*
	Mitarbeit und Engagement	Kodiere alle Stellen, in denen die Interviewten darüber sprechen, welche Faktoren Einfluss nehmen auf die Bereitschaft zur Mitarbeit der einzelnen Teilnehmenden im Netzwerk oder wie die Mitwirkbereitschaft den Verlauf des Projekts beeinflusst. Hinweis 1: wahrgenommener Nutzen als Einflussfaktor wird als „Mehrwert“ kodiert	*„Schulsozialarbeit, Ganztagbereich, die KollegInnen, die da arbeiten, die brauchen was, was man gleich umsetzen kann und sparen sich diesen theoretischen Aufbau.“*
	Arbeitskulturen	Kodiere alle Stellen, in denen die Interviewten darüber sprechen, wie die Arbeitskulturen oder arbeitsbezogene Praktiken Einfluss nehmen auf die Arbeit im Netzwerk	*„Dass es auch AG-Mitglieder gibt, die eher koordinative Aufgaben oder steuernde Aufgaben haben und nicht direkte Arbeit mit den/Das ist einerseits gut, aber andererseits erhöht das immer die Flughöhe bei der Betrachtung und Besprechung von Themen und das, wir wollen ja hauptsächlich in diesem Rahmen konkret arbeiten und konkret sein.“*
	Teilnehmerfeld	Kodiere alle Stellen, in denen die Interviewten darüber sprechen, welche Teilnehmenden für den Erfolg des Projekts relevant sind bzw. wie der Kreis der Teilnehmer*innen Einfluss auf die Arbeit des Netzwerks nimmt	*„… die Schulen sind nun mal so ein Baustein in der Präventionskette, der leider nicht so eingebunden ist, der auch schwierig einzubinden ist, weil Schulleiter das auch so nicht kennen und da auch vielleicht nicht so die Ressourcen für bereitstellen wollen, was aber enorm wichtig ist.“*
	Mehrwert	Kodiere alle Stellen, in denen die Interviewten darüber sprechen, welche wahrgenommenen Nutzen relevant sind für ein Engagement der Teilnehmer*innen im Netzwerk	„Interessierte Mitarbeiter*innen, die neu in die Einrichtungen nach Freiham kommen, man sucht intuitiv nach Vernetzung, nach einem Punkt in dem Sozialraum, wo man auch andere kennenlernt und je mehr man in eigenen Konzepten Vernetzung hat, desto attraktiver ist es.“

## Ergebnisse

### Studienteilnehmer*innen

Mit dem Netzwerkmanagement der Präventionskette Freiham wurden von April 2020 bis Oktober 2021 insgesamt 6 Interviews geführt. Auf der Position des Netzwerkmanagements erfolgte im Sommer 2020 ein personeller Wechsel. Die ersten beiden Interviews wurden mit der bis Sommer 2020 für das Netzwerkmanagement verantwortlichen Person geführt, die weiteren 4 mit dem/der Nachfolger*in auf dieser Position.

Die Fokusgruppe mit Mitgliedern der Begleitgruppe fand im Februar 2021 statt. Insgesamt nahmen 4 Personen teil. Das Sozialreferat der Stadt München war mit 2 Mitarbeiter*innen vertreten, das Gesundheitsreferat und das Referat für Bildung und Sport mit je einer/einem Vertreter*in.

Die Interviews mit lokalen Fachkräften erfolgten in einer ersten Erhebungswelle von Oktober bis Dezember 2020 und in einer zweiten Erhebungswelle von Juni bis Juli 2021. Insgesamt wurden 19 Interviews (Welle 1: 10; Welle 2: 9) mit 12 Teilnehmer*innen geführt: 7 Personen wurden in beiden Wellen interviewt, 3 nur in Welle 1 und 2 nur in Welle 2. Ein*e Teilnehmer*in aus Welle 1 zog ihre Einverständniserklärung nach Lesen seines/ihres Transkripts zurück, sodass insgesamt 18 Interviews von 11 Teilnehmer*innen für die Datenauswertung herangezogen wurden. Bei den Teilnehmer*innen waren die Sektoren Bildung und Soziales am stärksten vertreten. Hinsichtlich altersgruppenspezifischer Arbeitsschwerpunkte ließen viele Teilnehmer*innen sich keiner festen Kategorie zuordnen, da sie mit beiden Zielgruppen arbeiteten (Tab. 2).

**Tab. 2:** Anzahl der lokalen Fachkräfte nach altersgruppenspezifischen Arbeitsschwerpunkten und Sektor, deren Interviews für die Analyse verwendet wurden

Sektor	Altersgruppenspezifische Arbeitsschwerpunkte			Gesamt
	0–6 Jahre	6–17+ Jahre	Übergreifend	
*Bildung*	1	3	–	4
*Soziales*	–	1	3	4
*Gesundheit*	2	–	–	2
*Übergreifend*	–	–	1	1
*Gesamt*	3	4	4	11

### Einflussfaktoren der Implementierung

**Abb. 2:**
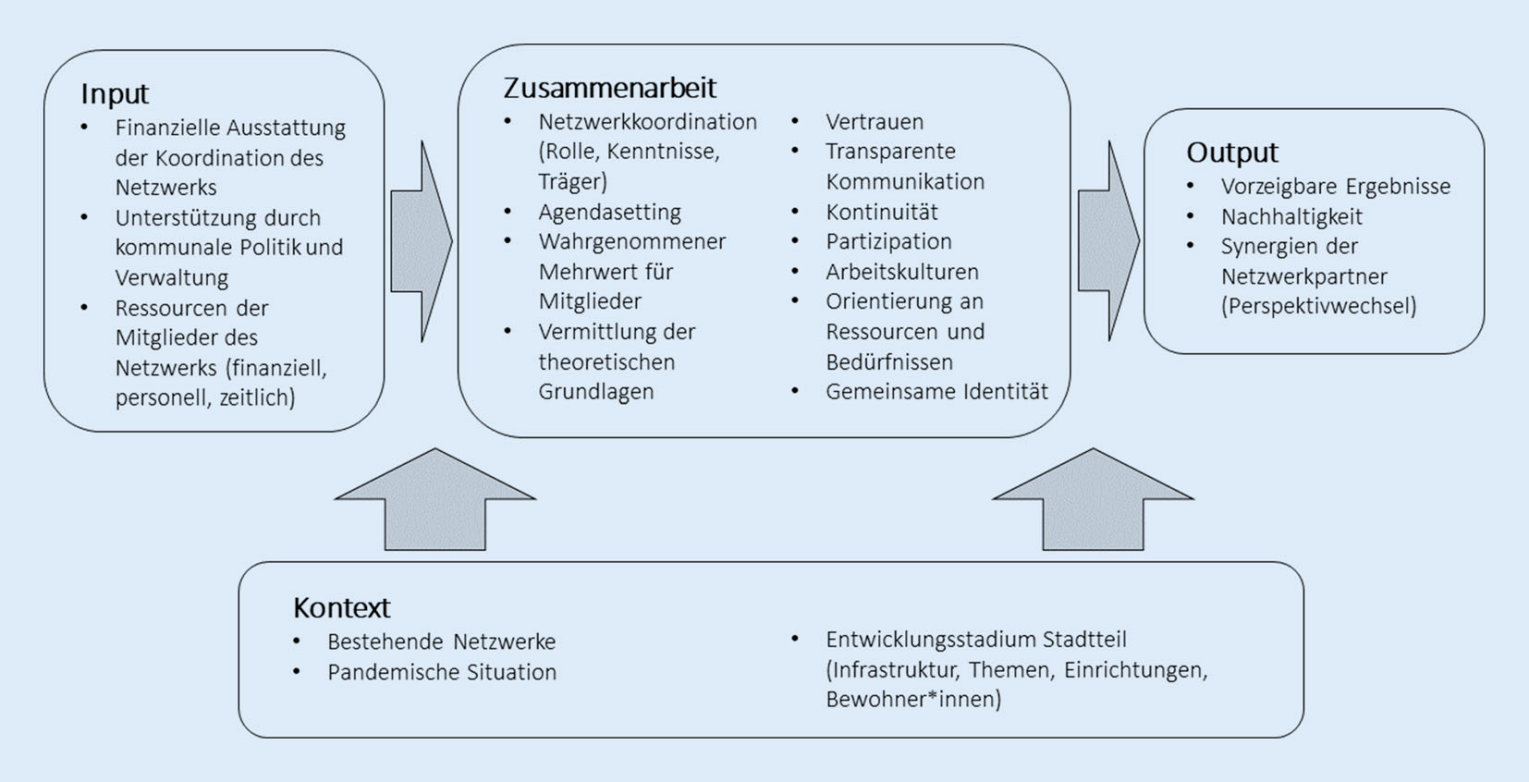
Einflussfaktoren für die Implementierung von Präventionsketten in Neubaugebieten

Die Darstellung der identifizierten Einflussfaktoren orientiert sich im Folgenden am „Bergen Model of Collaborative Functioning“ [4, 5], einem Framework zur Analyse von Zusammenarbeit speziell im Rahmen von Gesundheitsförderung. Das Modell unterteilt relevante Faktoren von Netzwerken in vier Bereiche: Input, Zusammenarbeit, Output und Kontext. Eine grafische Übersicht der identifizierten Einflussfaktoren ist in Abb. 2 zu sehen.

#### Input

Die befragten Teilnehmer*innen sahen eine ausreichende finanzielle Ausstattung als entscheidenden Einflussfaktor für den Erfolg des Netzwerkaufbaus an. Im Fall der Präventionskette Freiham war die langfristige Finanzierung des Netzwerkmanagements als zentraler Akteur für die Implementierung im Stadtteil zum Zeitpunkt der Datenerhebungen unklar. Es wurde von Befragten vermutet, dass diese Ungewissheit sich hemmend auf die Bereitschaft zum Engagement im Netzwerk auswirke. Zudem wurde darauf hingewiesen, dass der enge finanzielle Rahmen auch das Zeitbudget des Netzwerkmanagements und damit die Möglichkeiten zur Aufbauarbeit begrenze.

„Wichtig ist, dass die Präventionskette weiterarbeiten kann, dass einfach auch die Sicherheit bekommt, die finanzielle Sicherheit. Dann wird wahrscheinlich die Haltung ein bisschen anders sein.“

„Davon hängt es wahrscheinlich auch bei vielen so ein bisschen das ab, wie viel Energie und Zeit steckt man in diese Vernetzung, wenn es vielleicht nach einem Jahr, nächstes Jahr oder wie, dann doch nicht weiter finanziert wird, hält man sich wahrscheinlich ein bisschen zurück.“

Auch ein Mangel an Ressourcen auf Seiten der Akteur*innen des Produktionsnetzwerks wurde als Hindernis beim Aufbau des Netzwerks genannt. Hier waren neben der Verfügbarkeit von finanziellen Mitteln die personelle Ausstattung und, damit verbunden, der Faktor Zeit zentrale Faktoren. Zum Zeitpunkt der Interviews waren überwiegend Einrichtungen im Nachbarstadtteil Neuaubing für das Gebiet Freiham zuständig. Das begrenzte die verfügbaren Kapazitäten für den neu entstehenden Stadtteil.

Eine Unterstützung für die Umsetzung der Präventionsstrategie in der kommunalen Verwaltung sowie von politischen Entscheidungsträger*innen nannten die Fachkräfte in den Interviews und in der Fokusgruppe aus mehreren Gründen als wichtigen Einflussfaktor, v. a. zur Sicherung der Finanzierung des Projekts.

„Ich denke, immer wichtig für Gelingen von solchen Projekten ist die Politik im Grunde genommen. Wir kriegen über den Stadtrat unser Geld, wir kriegen über den Stadtrat unsere Stellen.“

Außerdem galt, aufgrund der hierarchischen Strukturen in der Verwaltung, der Rückhalt von Politik und Referatsspitzen als wichtiges Signal für die nachgeordneten Ebenen innerhalb der Behörden für ein Engagement. Gleichzeitig waren die Verantwortlichen in den Referaten gefragt, ihren Fachkräften zeitliche Kapazitäten zur Mitarbeit im Netzwerk zur Verfügung zu stellen.

#### Zusammenarbeit

Als zentralen Faktor für das Gelingen der Zusammenarbeit im Netzwerk nannten die Teilnehmer*innen die Position der zentralen Netzwerkkoordination – im Falle der Präventionskette Freiham des Netzwerkmanagements. Dieses habe die Aufgaben, als Ansprechpartner für die Einrichtungen zu relevanten Themen im Stadtteil zu dienen, für die Bearbeitung dieser Themen zu sorgen, die richtige Agenda zu setzen und die Teilnahmebereitschaft der beteiligten Akteur*innen zu fördern. Gute Kenntnisse über den Stadtteil, die relevanten Personen und die Arbeitsprozesse in der Kommune wurden ebenfalls als wichtige Eigenschaften angesehen. Eine Bedeutung wurde dabei nicht nur der Person und der Tätigkeit des Netzwerkmanagements zugesprochen, sondern auch der Institution, der die dafür verantwortlichen Personen entstammen. So wurde es als Vorteil angesehen, dass MAGs bei der Präventionskette Freiham das Netzwerkmanagement stellt – ein Träger, der selbst keine Einrichtung im Stadtteil betreibt und deswegen von anderen Akteur*innen eher als neutrale Instanz angesehen werden könne. Die Rolle des Netzwerkmanagements als zentrale Ansprech- und Koordinationsstelle wurde als wesentlich für die Verstetigung der Arbeit in der Präventionskette angesehen, sodass bei einem Wegfall der Position das Ende des Netzwerks befürchtet wurde.

„Das ist wie in jedem Netzwerk, wenn es keine Struktur gibt, die adressierbar ist und die Themen im Blick hat, wenn die wegfällt, dann schläft das Netzwerk über kurz oder lang zwangsläufig ein. Das weiß man einfach, dass das langfristig nicht läuft.“

Den Wechsel in der Position des Netzwerkmanagements im Sommer 2020 erlebten die Befragten dagegen nicht als Bruch. Aus ihrer Sicht gelang der Übergang ohne Schwierigkeiten und hatte keinen negativen Einfluss auf den Implementierungsprozess. Allerdings merkte eine Fachkraft an, dass solche Personalwechsel immer kritisch beäugt würden.

Für ein Engagement der Fachkräfte im Netzwerk wurden wechselseitige Sympathie, Vertrauen, eine offene Kommunikation, das Gefühl der Wertschätzung und die Herausbildung einer gemeinsamen Identität als wesentliche Faktoren genannt. Hier stellte das Setting des neu entstehenden Stadtteils eine Herausforderung dar, da viele Akteur*innen sich nicht kannten. In der Anfangsphase des Netzwerkaufbaus musste deswegen viel Zeit in das gegenseitige Kennenlernen und das Vorstellen von einzelnen Einrichtungen investiert werden. Ein individueller Nutzen hatte für die Fachkräfte ebenfalls einen positiven Einfluss auf die Bereitschaft zur Teilnahme am Netzwerk. Hier wurden die Versorgung mit Informationen, insbesondere aus der Stadtverwaltung, die Vernetzung mit anderen Akteur*innen und die Verfügbarkeit eines Ansprechpartners bei auftretenden Herausforderungen als relevante Faktoren angesehen. Hinsichtlich der Herausarbeitung der Vorteile spielten dabei die Vielfalt der Akteur*innen mit ihren jeweiligen Arbeitskulturen und Interessen, die miteinander vereinbart werden müssten, eine Rolle.

„Dass, vor allem, wenn es auch so viele Teilnehmende aus unterschiedlichen Bereichen gibt, das hinzubekommen, dass alle irgendwie am Ball bleiben und alle für sich auch gute Ergebnisse oder gute, was auch immer, Informationen oder Kenntnisse mit rausziehen.“

Gerade die Einbindung von Schulen in das Netzwerk gelang im Untersuchungszeitraum nach Ansicht der Befragten nur unzureichend. Das wurde zum einen mit der Arbeitskultur in Schulen erklärt, in der Netzwerkarbeit noch nicht verankert sei. Zudem wurden organisatorische Hürden und Ressourcenknappheit als Barrieren für ein stärkeres Engagement von Verantwortlichen aus Schulen vermutet. Als Hindernis für die Vernetzung wurde zudem mangelnde Kontinuität bei der Teilnahme auf Seiten der Akteur*innen genannt.

Mehrere Teilnehmer*innen betonten eine partizipative Vorgehensweise als wichtigen Erfolgsfaktor bei der Zusammenarbeit. Diese müsse sich an den Bedürfnissen und verfügbaren Ressourcen der Mitglieder im Netzwerk orientieren und außerdem die Zielgruppen der Kinder und Familien im Stadtteil bei der Planung mit einbeziehen.

„… dass wir das partizipativ gestalten und schauen, aufzugreifen, was vorhanden ist an Interessen, an Ressourcen, an Möglichkeiten, weil ich nur so glaube, dass ein Netzwerk auch nachhaltig über einen längeren Zeitraum funktionieren kann. Das bezieht sich aber auch auf die Ebene der Zielgruppen, der Kinder, Jugendlichen und Familien. Dass wir von Anfang an darauf achten, dass wir partizipativ diese Zielgruppen mitdenken und das mit einbinden in die Arbeit.“

Gegensätzliche Ansichten gab es zur Frage, inwiefern theoretische Konzepte der Präventionskette vermittelt werden müssten. Einerseits wurde die Notwendigkeit gesehen, die Fachkräfte mit diesen Konzepten vertraut zu machen, da eine Präventionskette in der Stadt München neu sei. Andererseits wurde angemerkt, dass dieser theoretische Überbau für viele Akteur*innen nicht relevant sei und der Fokus auf der praktischen Arbeit liegen müsse.

„Schulsozialarbeit, Ganztagbereich, die Kolleg*innen, die da arbeiten, die brauchen was, was man gleich umsetzen kann und sparen sich diesen theoretischen Aufbau.“

#### Output

Teilnehmer*innen betonten die Bedeutung von nachhaltiger Projektarbeit, die außerdem einen echten Mehrwert für die Zielgruppen bieten müsse. Als positiver Synergieeffekt wurde hier die Beteiligung von Einrichtungen aus verschiedenen Bereichen in der Präventionskette genannt, da sie den einzelnen Mitgliedern einen Perspektivenwechsel erlaube und die damit entstehenden Produkte eine höhere Effizienz und Praxisnähe erreichen könnten. Mehrere Fachkräfte stellten die Notwendigkeit heraus, konkrete Projekte aus der Netzwerkarbeit zu entwickeln, um einerseits gegenüber Verwaltung und Politik vorzeigbare Produkte zu besitzen und andererseits intern v. a. den zeitlichen Ressourcenaufwand zu rechtfertigen.

„Idealerweise, in meiner Idealvorstellung, trifft man sich eben nicht um des sich Treffens willens, um sich gegenseitig zu erzählen, was gerade gut oder was gerade nicht so gut läuft, sondern um an Lösungen gemeinsam zu arbeiten.“

Als Herausforderung bei der Schaffung von Output wurde genannt, dass im Neubaugebiet zum Zeitpunkt der Interviews nur wenige Bewohner*innen lebten. Dadurch sahen Teilnehmer*innen einen Mangel an konkreten Aufgaben, denen das Netzwerk sich widmen könne, verbunden mit der Gefahr von Aktionismus, aus dem nicht sinnvolle oder nachhaltige Projekte resultieren könnten.

„Ein Punkt ist natürlich, wenn vor Ort Menschen schon leben würden, dann könnte man sich auf die Themen stürzen und damit arbeiten und das, ja, weiß ich nicht, wie ich das korrekt formulieren soll, aber wenn noch keine wirklichen Probleme, Herausforderungen vor Ort vorhanden sind, dann gerät man vielleicht in Versuchung auch welche zu schaffen oder zu suchen.“

#### Kontext

Mehrere Kontextfaktoren beeinflussten nach Aussagen der Teilnehmer*innen den Prozess der Implementierung der Präventionskette im Stadtteil. Zum Zeitpunkt der Interviews befand sich der Stadtteil Freiham in einem frühen Entwicklungszustand. Deswegen wohnten nur wenige Bewohner*innen im Gebiet und es bestand wenig unterstützende Infrastruktur für Familien. Der Großteil der Einrichtungen, in denen die Fachkräfte tätig waren, war noch nicht in eigenen Räumen vor Ort präsent, sondern agierte von angrenzenden Stadtteilen aus. Das begrenzte die verfügbaren Kapazitäten für den neuen Stadtteil und erschwerte den Zugang zu Bewohner*innen. Einige geplante Umzüge von Einrichtungen nach Freiham verzögerten sich deutlich aufgrund von baulichen Herausforderungen. Das Fehlen von Infrastruktur wurde neben dem Fehlen von Bewohner*innen und damit verbundenen konkreten Aufgaben als Hindernis für die inhaltliche und praktische Ausgestaltung des Netzwerks angesehen.

Als Hindernis für eine breite Unterstützung in der kommunalen Verwaltung wurde wahrgenommen, dass die Präventionskette in einer Großstadt wie München nur eines von vielen Projekten in der Stadtverwaltung sei und als langfristiges Präventionskonzept dort von aktuell dringlicheren Herausforderungen – wie der COVID-19-Pandemie zum Zeitpunkt der Interviews – in den Hintergrund gedrängt werden könnte. Zudem wurde eine hohe Erwartungshaltung als negativer Einflussfaktor genannt, die den Rückhalt in das Projekt schwinden lassen könnte.

„Andere Stolperfalle ist, vor Ort, glaube ich auch, die Schlüsselpersonen in der lokalen Politik und so weiter, die Erwartungen haben, die die Präventionskette nicht erfüllen kann und das zu einem Bumerang werden kann, aus meiner Sicht. Also, wenn da dann ein Problem vorliegt, dann soll die Präventionskette das lösen.“

Ein weiterer relevanter Kontextfaktor waren bereits bestehende Netzwerkstrukturen. Die Stadt München verfügt mit REGSAM (Regionales Netzwerk für Soziale Arbeit in München) bereits über ein etabliertes Netzwerk, das auch im Stadtgebiet von Freiham tätig ist. Hier zeigte sich von Seiten der Präventionskette Freiham die Notwendigkeit, lokalen Fachkräften den Unterschied zwischen den Netzwerken klarzumachen. Gleichzeitig gab es Abklärungsbedarf mit den zuständigen Fachkräften des REGSAM-Netzwerks, um konkurrierende Parallelstrukturen zu vermeiden. Innerhalb des Studienzeitraums ergab sich die Lösung, dass die Präventionskette Freiham als offizieller Facharbeitskreis für Kinder, Jugend und Familien innerhalb des REGSAM-Netzwerks für den Stadtteil auftreten solle.

Eine spezifische Barriere bei der Implementierung des Netzwerks der Präventionskette Freiham war zudem der Kontext der COVID-19-Pandemie, die nahezu gleichzeitig mit Intensivierung des lokalen Netzwerkaufbaus begann und über den gesamten Studienzeitraum andauerte. Aufgrund von Kontaktbeschränkungen fanden Treffen der Arbeitsgemeinschaften während des Untersuchungszeitraums überwiegend in digitalen Räumen statt. Die interviewten Fachkräfte stimmten weitgehend überein, dass die Abwesenheit von Präsenztreffen das gegenseitige Kennenlernen, den Kontaktaufbau und die produktive Projektarbeit erschwert hätte.

„Aber, dass man die Kontakte pflegt, sich austauscht in Nebengesprächen, vor und nach dem Treffen und in den Pausen und so weiter. Das finde ich eigentlich, das hat halt komplett gefehlt.“

„Ich sage mal so, das sind auch erschwerte Bedingungen, das gilt auch hier. In einer großen Runde, online, wirklich etwas zu entwickeln. Also, ich sage mal, Projektentwicklung hat auch immer irgendwie was mit einem kreativen Arbeitsprozess zu tun, aus meiner Sicht und das, finde ich, ist online total schwierig abzubilden (…)“

## Diskussion

In unseren qualitativen Daten konnten wir zahlreiche Einflussfaktoren für einen gelingenden Aufbau von Präventionsketten-Netzwerkstrukturen in Neubaugebieten identifizieren. Auf der Ebene des Inputs waren eine ausreichende und langfristige finanzielle Ausstattung des Netzwerks sowie Rückhalt in Politik und Verwaltung wichtige Faktoren. Für die lokalen Einrichtungen waren die Verfügbarkeit von finanziellen, personellen und zeitlichen Ressourcen entscheidend, um sich im Netzwerk engagieren zu können. Auf der Ebene der Zusammenarbeit waren die Fähigkeit der zentralen Netzwerkkoordination zur richtigen Themensetzung, Förderung der Motivation und Management der Diversität an Partner*innen im Netzwerk relevante Faktoren. Für die einzelnen Netzwerkmitglieder waren eine vertrauensvolle Zusammenarbeit, eine transparente Kommunikation und die Wahrnehmung eines konkreten Nutzens aus der Arbeit entscheidend. Beim Output wurde die Bedeutung von einerseits vorzeigbaren, gleichzeitig auch nachhaltigen und sinnvollen Ergebnissen der Netzwerkarbeit betont. In Bezug auf den Kontext erwies sich der Umgang mit bereits vorhandenen Netzwerkstrukturen als Herausforderung.

Die oben genannten Einflussfaktoren beziehen sich auf die Implementierung von Präventionsketten unabhängig vom Entwicklungszustand des Stadtteils. Als spezifisch für das Setting eines Neubaugebiets stellten sich das Fehlen von Infrastruktur, Unsicherheit hinsichtlich der Bedarfe und Bedürfnisse zukünftiger Bewohner*innen sowie der Fakt, dass viele Fachkräfte noch nicht vor Ort waren oder dem Stadtteil nur begrenzt Aufmerksamkeit schenken konnten, als relevante Faktoren heraus.

Dabei ist herauszustellen, dass die von uns identifizierten Faktoren jeweils das Potenzial haben, ein Erfolgsfaktor oder eine Barriere darzustellen. Wird z. B. die kommunale Gesamtstrategie durch politische Entscheidungsträger*innen unterstützt, kann das für die Umsetzung förderlich sein. Fehlt die Unterstützung hingegen, kann dies die Implementierung erschweren.

### Vergleich mit bisher erschienener Literatur

#### Input

Die Ergebnisse dieser Studie stimmen weitgehend mit früheren Untersuchungen zu Präventionsketten und vergleichbaren sektorenübergreifenden kommunalen Netzwerken zur Prävention und Gesundheitsförderung im Kinder- und Jugendbereich überein. Eine Interviewstudie von Sowarka und Coenen mit Verantwortlichen von Präventionsketten in deutschen Kommunen [32] sowie eine Interviewstudie von Bentgens zu Akteur*innen aus intersektoralen Netzwerken in der Kinder- und Jugendhilfe in Düsseldorf [1] kamen ebenfalls zu dem Ergebnis, dass verfügbare finanzielle Ressourcen auf Seiten des Netzwerks sowie seiner Mitglieder ein essentieller Gelingensfaktor sind. Der Zwischenbericht zum Präventionsnetzwerk Ortenaukreis betont zudem die Bedeutung der Ressource Zeit für die Projektleitung [14], damit diese aktiv gestalten kann. Ein Punkt, der auch von unseren Teilnehmer*innen wiederholt aufgebracht wurde.

Die Bedeutung des politischen Rückhalts wurde bei Sowarka und Coenen ebenso wie in unserer Studie hervorgehoben [32]. Die Begleitforschung zum Projekt „Kommunale Präventionsketten“ (ehemals „KeKiz“, „Kein Kind zurücklassen“) in NRW ergab zudem bürokratische Strukturen als hindernde Faktoren. Darüber hinaus stellte sie die Notwendigkeit eines Mandats von der Führungsebene in der Verwaltung als wesentlich dar, um Veränderungen bewirken zu können [3]. Ein Hindernis, auf das Präventionsketten und vergleichbare Projekte zur Prävention und Gesundheitsförderung stoßen, ist der in der Regel enge finanzielle Handlungsspielraum von Kommunen, der eine Förderung erschwert [2, 33]. Hier besteht das bekannte Problem von Maßnahmen im Bereich der Gesundheitsförderung im kommunalen Setting, dass der Nutzen erst nach langer Zeit deutlich wird, der Aufbau entsprechender Strukturen aber unmittelbare Kosten verursacht. Belege der Effektivität von sektorenübergreifender Präventionsarbeit aus früheren Modellversuchen können als überzeugende Argumente für die Spitzen in Verwaltung und Politik dienen. Trotz erster positiver Hinweise sind diese allerdings noch rar [2].

#### Zusammenarbeit

Auf der Ebene der Zusammenarbeit zeigt die bisherige Forschung ebenfalls die Bedeutung von Fähigkeiten der Netzwerkkoordination zur Steuerung des Aufbau- und Arbeitsprozesses als wesentlichen Einflussfaktor. In der Interviewstudie von Sowarka und Coenen wurden die für die Netzwerkkoordination Verantwortlichen als zentrales Bindeglied zwischen den einzelnen Einrichtungen und der Verwaltung genannt [32]. Hier wurde zudem als wichtig angesehen, dass die Netzwerkkoordination glaubwürdig eine neutrale Position zwischen den Akteur*innen einnehmen könne. So sei es aufgrund kurzer Dienstwege von Vorteil, wenn die entsprechenden Personen der Verwaltung angegliedert sind, gleichzeitig aber nichts selbst Teil der Verwaltung sind, da sie sonst weisungsgebunden auftreten müssten. Bei der Evaluation des Projekts „Kommunale Präventionsketten“ in NRW zeigte sich kein eindeutiger Vorzug einer bestimmten Form, wie die Koordinationsstelle in die Behördenstruktur eingegliedert ist. Entscheidend sei eine Adaptation an die jeweiligen Gegebenheiten [3]. Eine nicht erfolgende Verstetigung der zentralen Koordination wurde hier, kongruent mit unseren Ergebnissen, als zentrales Hindernis für eine erfolgreiche Netzwerkarbeit angesehen.

Die bisherige Forschung zu Präventionsketten stimmt weitgehend überein, dass es zentrale Aufgabenfelder der Netzwerkleitung sind, die Arbeitsweise des strategischen Ansatzes den Akteur*innen zu verdeutlichen, den Mehrwert zu vermitteln, die Vielfalt der Akteur*innen zu managen und transparent zu kommunizieren [1, 3, 14, 32]. Ein Review von Corbin et al. zu Erfolgsfaktoren intersektoraler Kooperationen in der Gesundheitsförderung betont die Bedeutung von Tätigkeiten wie Kommunikation und Evaluation, die nicht direkt mit den Projektzielen im Zusammenhang stehen, aber erheblich zur Aufrechterhaltung der Netzwerke beitragen [4]. Bentgens beschreibt als wichtige Eigenschaften der Kommunikation im Netzwerk, dass sie sachlich, umfänglich, auf Augenhöhe, regelmäßig und persönlich ablaufen müsse [1].

Dem Setting Schule kommt in der kommunalen Gesundheitsförderung von Kindern und Jugendlichen eine besondere Bedeutung zu [10]. Analog zu unseren Ergebnissen, hat sich bei früheren intersektoralen Ansätzen gezeigt, dass die Kooperation zwischen Schulen und anderen Bereichen oft schwierig ist [1, 3, 9]. Eine Befragungsstudie an dänischen Schulen zu Erfahrungen mit sektorenübergreifender Zusammenarbeit legt nahe, dass es Schulen schwerfällt, Gesundheitsförderung in ihre Praxis zu integrieren und diese meist als zusätzliche Belastung wahrgenommen wird [31]. Integrierte Ansätze zur Gesundheitsförderung sollten daher einerseits den Fokus von Schulen auf ihre Kernkompetenz Bildung berücksichtigen und andererseits an Schulen ein Bewusstsein für die Bedeutung von Gesundheitsförderung und Kooperation schaffen [31].

#### Output

Erfolg ist in der kommunalen Gesundheitsförderung ein schwer bestimmbarer Faktor, da Effekte in der Regel erst nach einem längeren Zeitraum zu beobachten und selten direkt auf die Maßnahmen zurückführbar sind [15]. Für die Verantwortlichen entsprechender Interventionen kann es eine Herausforderung darstellen, intern und extern die Sinnhaftigkeit des Projekts darzustellen. Befragte in unserer Studie betonten daher die Wichtigkeit von konkretem Output. In der Begleitforschung zu „Kommunale Präventionsketten“ in NRW wurde ebenfalls darauf verwiesen, dass entsprechende vorzeigbare Ergebnisse ein wichtiges Argument für ein Engagement im Netzwerk seien [3]. Das Bedürfnis, Erfolge präsentieren zu können, kann allerdings in Konflikt treten mit einer nachhaltigen Projektarbeit, wenn es für die komplexen Ursachen von gesundheitlicher Ungleichheit keine schnellen Lösungen gibt. Hier scheint es sinnvoll, intern und extern diese Problematik zu thematisieren und die Erwartungshaltung den realen Gegebenheiten anzupassen.

#### Kontext

In Bezug auf grundsätzliche Einflussfaktoren für den Aufbau von Präventionsketten stimmt unsere Studie weitgehend mit Ergebnissen bisheriger Forschung überein. Neue Erkenntnisse liefert sie in Bezug auf das spezielle Setting eines neuen Stadtteils, in dem die Implementierung der Präventionskette Freiham stattfindet. Teilnehmer*innen sahen es als eine Herausforderung an, ein produktives Netzwerk mit entsprechendem Output aufzubauen, während es aufgrund des Entwicklungszustands des Stadtteils ein Mangel an Fachkräften, Bewohner*innen und damit verbundenen konkreten Aufgaben gab. Verantwortliche müssen hier einerseits die Erwartungshaltung von Politik und Verwaltung managen, und andererseits die Netzwerkmitglieder trotz langsamen Starts zur langfristigen Teilnahme motivieren. Die Begleitforschung zum Projekt „Kommunale Präventionsketten“ fand zudem die große Anzahl an Problemen und Aufgaben speziell in Großstädten als eine Herausforderung, gegen die einzelne Projekte sich durchsetzen müssten [3], ein Umstand, der auch in unserer Studie als Barriere genannt wurde.

Ein weiterer Kontextfaktor war die COVID-19-Pandemie, die den vollständigen Zeitraum des Erhebungszeitraums prägte. Diese erschwerte den Netzwerkaufbau, indem einerseits Fachkräfte mit ihren Ressourcen anderweitig eingebunden waren und andererseits die Netzwerktreffen in digitalen Räumen stattfinden mussten, was Gruppenbildungsprozesse und Projektarbeit beeinträchtigte. Während COVID-19 für zukünftige Projekte möglicherweise keine oder nur eine untergeordnete Rolle spielt, betonen unsere Ergebnisse die Bedeutung von direkter und persönlicher Kommunikation in der Netzwerkarbeit, die bereits frühere Forschungen zeigte [1, 5]. Inwiefern auch nach der Pandemie in der kommunalen Gesundheitsförderung Treffen in digitale Räume verlegt werden, ist zum jetzigen Zeitpunkt nicht absehbar.

### Stärken und Limitationen

Diese Arbeit hat mehrere Stärken. Die Durchführung der Studie und die Auswertung der Daten wurden, soweit möglich, gemäß den COREQ-Guidelines („Consolidated criteria for reporting qualitative research“) für qualitative Forschung berichtet [34]. Die Analyse erfolgte durch 3 Personen im Forschungsteam. Konflikte wurden gemeinsam geklärt, um eine hohe Übereinstimmung zwischen Kodierer*innen sicherzustellen. Mit dem Netzwerkmanagement, den Referatsmitarbeiter*innen in der Begleitgruppe sowie den lokalen Fachkräften wurden Teilnehmer*innen aus unterschiedlichen Positionen des Netzwerks rekrutiert, um ein umfassendes Bild des Netzwerksaufbaus zu erhalten. Teilnehmer*innen waren z. T. seit Jahren, z. T. seit wenigen Monaten und in einem Fall noch gar nicht im Netzwerk engagiert. Bei der Rekrutierung der Fachkräfte wurde zudem darauf geachtet, möglichst alle Altersstufen in Bezug auf die Zielgruppen und alle Sektoren zu erfassen.

Diese Studie bestätigt in weiten Teilen frühere Untersuchungen zu relevanten Einflussfaktoren bei der Einrichtung von Präventionsketten und erweitert sie um Aspekte, die sich auf die Implementierung im Kontext eines im Aufbau befindlichen Stadtteils beziehen. Zudem wurden die Interviews und die Fokusgruppe zu einem frühen Zeitpunkt der Implementierung durchgeführt. Dadurch geben unsere Ergebnisse Rückschluss darauf, welche Faktoren gerade zu Beginn eines entsprechenden Projekts von den Verantwortlichen berücksichtigt werden sollten.

Diese Studie hat mehrere Limitationen. Trotz der *Purposeful-sampling*-Strategie bei der Rekrutierung der Fachkräfte im Stadtteil konnten einige Bereiche nur unzureichend berücksichtigt werden. So wurde nur eine Fachkraft aus dem Gesundheitssektor interviewt. Diese Einschränkung lässt sich zum einen damit erklären, dass es zum Zeitpunkt der Erhebungen nur wenig Personal aus diesem Sektor gab, welches schwerpunktmäßig im Stadtteil tätig war. Zudem bedeutete die COVID-19-Pandemie für viele Menschen im Gesundheitssektor eine erhebliche Belastung [13], was vermutlich ein Hindernis für die Teilnahmebereitschaft an dieser Studie war. In einem Fall begründete eine für eine Teilnahme angefragte Person ihre Absage explizit mit der Arbeitsbelastung durch die laufende Pandemie.

Zwischen dem Forschungsteam und Verantwortlichen der Präventionskette Freiham bestand während des Untersuchungszeitraums ein regelmäßiger Austausch. Zudem waren viele Befragte persönlich miteinander bekannt. Mit den Mitgliedern der Begleitgruppe wurden nur Akteur*innen aus der Stadtverwaltung rekrutiert, die bereits seit Jahren mit dem Projekt vertraut waren und diesem positiv gegenüberstanden. Diese Faktoren könnten den Prozess der Datenerhebung beeinflusst haben und dazu geführt haben, dass Teilnehmer*innen tendenziell erwünschte Antworten gaben. Da für die Studie ein relativ kleiner Personenkreis befragt wurde, wurde aus Datenschutzgründen bei der Darstellung der Ergebnisse auf eine nähere Beschreibung der Personen hinter den einzelnen Zitaten und Aussagen verzichtet.

Aufgrund der Kontakteinschränkungen während der COVID-19-Pandemie wurden der Großteil der Interviews sowie die Fokusgruppe digital durchgeführt. Das könnte ebenfalls die Inhalte und die Qualität unserer Datenerhebungen beeinflusst haben. Allerdings ist es in der bislang erschienenen Literatur unklar, inwiefern die Ergebnisse qualitativer Forschung in digitalen Formaten sich gegenüber Erhebungen in Präsenz unterscheiden [17, 30, 36]. Zudem ist unklar, inwiefern sich Erkenntnisse aus der Zeit vor der COVID-19-Pandemie auf die Zeit während und nach der Pandemie übertragen lassen.

Als weitere wesentliche Einschränkung beeinflusste der Hintergrund der COVID-19-Pandemie den Aufbau des Netzwerks in Freiham in erheblichem Maße. Daher ist es fraglich, inwiefern sich die Ergebnisse unserer Studie auf andere Kontexte übertragen lassen, eine Frage, die bei kommunalen Interventionen ohnehin schwer zu beantworten ist [12]. Zudem hatte das frühe Stadium in der Implementierung des Netzwerks, in dem die Studie stattfand, auch Nachteile. Einflussfaktoren für die konkrete Zusammenarbeit im Netzwerk stützen sich deswegen v. a. auf Vermutungen der Befragten, weniger auf praktische Erfahrungen.

## Schlussfolgerungen

Der Aufbau von intersektoralen Netzwerken zur Gesundheitsförderung von Kindern und Jugendlichen in neu entstehenden Stadtteilen ist eine Herausforderung. Die ausreichende Verfügbarkeit von Ressourcen auf Seiten der Verantwortlichen sowie der Partner*innen ist nach unseren Erkenntnissen essentiell für einen langfristigen Erfolg, ebenso der Rückhalt in Politik und Verwaltung. Ebenso entscheidend scheint es zu sein, dass es dem Netzwerkmanagement gelingt, die diversen Akteur*innen in den Arbeitsprozess einzubinden und den Beteiligten mit ihren Interessen jeweils einen Mehrwert zu bieten.

Das Setting eines Neubaugebiets innerhalb einer Großstadt bringt ebenfalls mehrere Herausforderungen mit sich. So bestand einerseits ein Mangel an Infrastruktur, Akteur*innen, Bewohner*innen und daraus resultierend ein Mangel an konkreten Aufgaben und an vorzeigbaren Erfolgen, die wichtig für den Rückhalt innerhalb und außerhalb des Netzwerks sind. Dieser Faktor sollte bei der Planung einer Präventionskette in entsprechenden Gebieten vermutlich besonders berücksichtigt werden, indem ausreichend Zeit und damit verbunden Ressourcen bereits in einem sehr frühen Stadium zur Verfügung gestellt werden. Gleichzeitig verfügen Kommunen bereits über etablierte Netzwerkstrukturen, sodass Partner*innen die Vorteile eines Präventionskettennetzwerks verdeutlicht und die Entstehung von parallelen Zuständigkeitsstrukturen vermieden werden müssen.

Unsere Forschung ist relevant für Kommunen, die die Implementierung einer Präventionskette planen – nicht nur, aber gerade dann, wenn der Aufbau in einem neu entstehenden Stadtgebiet erfolgen soll. Trotz aller Herausforderungen erscheint es ein sinnvolles Ziel, den integrativen Ansatz von Präventionsketten zur sektorenübergreifenden Zusammenarbeit relevanter Akteur*innen von Beginn an beim Aufbau kommunaler Unterstützungsstrukturen für Kinder, Jugendliche und Familien zu berücksichtigen, um gesundheitlichen Ungleichheiten entgegenzuwirken.

## Fazit für die Praxis

- Der vorliegende Beitrag berichtet über die erste Studie, die Einflussfaktoren zum Aufbau von Präventionsketten für das spezifische Setting eines Neubaugebiets untersucht.
- Für den erfolgreichen Aufbau ist eine langfristig angelegte Unterstützung durch politische Entscheidungsträger*innen und die kommunale Verwaltung essentiell. Die zentrale Netzwerkkoordination benötigt ausreichend finanzielle Ressourcen, um nachhaltige Strukturen aufbauen zu können.
- Potenzielle Mitglieder des Präventionskettennetzwerks müssen einen Mehrwert in ihrer Teilnahme an der Netzwerkarbeit sehen. In unserer Studie werden v. a. der Ausbau von Kontakten und die Versorgung mit Informationen als solche Mehrwerte angesehen.
- Für das Setting eines Neubaugebiets ist die Asynchronität von Vernetzungsprozessen und konkreten Aufgaben vor Ort eine besondere Herausforderung. Hier scheinen das Management der Erwartungshaltungen sowie eine nachhaltige Orientierung bei der Planung wichtig.
